# Health system strengthening and hypertension management in China

**DOI:** 10.1186/s41256-016-0013-8

**Published:** 2016-09-21

**Authors:** Kehui Huang, Yu Ting Song, Yong Huan He, Xing Lin Feng

**Affiliations:** School of Public Health, Peking University, Xueyuan road 38#, Beijing, 100191 People’s Republic of China

**Keywords:** Health system, Hypertension management, China

## Abstract

**Background:**

Non-communicable diseases are the leading causes of global burden of diseases, and hypertension is one of the most important risk factors. Hypertension prevalence doubled in China in the past decade and affects more than 300 million Chinese people. In the review we systematically searched peer-reviewed publications that link health system level factors with hypertension management in China and provide the current knowledge on how to improve a country’s health system to manage the hypertension epidemic.

**Methods:**

A framework was developed to guide the review. The database of PubMed, CNKI were systematically searched from inception to April 13, 2016. Two authors independently screened the searched results for inclusion, conducted data extraction and appraised the quality of studies. Key findings were described according to the framework.

**Findings:**

Five hundred seventy-two publications were identified, where 11 articles were left according to the inclusion and exclusion criteria. The study periods range from 2010 to 2015. All about 11 researches linked health system factors to the outcome of hypertension management. And the outcomes were just focused on the awareness, treatment and control of hypertension but not hypertension incidence. One study is about the role of health system governance, investigating the performance of different organized community health care centers; three studies were about health financing comparing differences in insurance coverage; three studies were about health information practicing the hypertension guidelines of China or the WHO, and the rest three about mechanisms of health service delivery. No researches were identified about physical resources for health and human resources for health.

**Conclusions:**

Hypertension prevalence has been rising rapidly in China and the management of hypertension in China is a detection problem rather than treatment problem. Limited evidence shows the positive effect of health system factors on hypertension management and joint efforts from health system and epidemiological researchers are warranted to extend knowledge in this area.

**Electronic supplementary material:**

The online version of this article (doi:10.1186/s41256-016-0013-8) contains supplementary material, which is available to authorized users.

## Background

Non-communicable diseases (NCDs)–including cardiovascular diseases (CVDs), cancer, diabetes, and chronic respiratory diseases–are the leading causes of global burden of diseases [1, 2], and are considered as “one of the major challenges for sustainable development in the 21st century” [3]. As one of the most important risk factors of CVDs, the pandemic of hypertension has become a global cause for concern [4]. With a prevalence rate of 40 % [1], it leads to 55 % of the global CVDs mortality burden and 7 % DALYs [5]. Hypertension could be managed well with low-tech, cost-effective and feasibly implemented primary care interventions, even in low resource settings [6, 7]. However, the proportions of hypertensive patients under appropriate control varies globally, and uncontrolled hypertension contribute to two-thirds of all strokes and half of all coronary diseases [8].

Confronted with the world crisis of NCDs, previous efforts were largely made in building innovative technologies and improving guidelines in researches and clinical practices [9, 10]. Only until recently, attentions have been drawn to the strengthening of a nations’ health system around the principle of universal health coverage [3]. According to the World Health Organization (WHO), efforts should be harmonized in strengthening a country’s governance structure in health, health financing, human resource for health, health information, health service delivery, and medicines and technologies from a system perspective in order to provide universal essential health care [11]. However, paucity of evidence was generated to answer the “how” question, particularly for the low-and middle-income countries (LMICs). For example, a recent systematic review conducted in the year 2013 identified 53 studies that analyzed the effects of health system strengthening efforts on hypertension awareness, treatment and control. Among these, only 11 were conducted in LMICs, where only one study was from China. However, this study did not explore any mechanism of health system strengthening efforts in improving hypertension management [12].

As the largest developing country, China bears a large burden of NCDs. Hypertension prevalence doubled in the past decade and affected around 337 million Chinese people in the year 2010 [13]. As a silent killer, 80 % of the CVDs mortality were estimated to be attributed to hypertension nationally, and more than half of those were premature death [14]. In this review, we systematically searched peer-reviewed publications that link health system level factors with hypertension management in the Peoples’ Republic of China. From the perspective of health system strengthening and population health management, we provide the current knowledge from China on how to improve a country’s health system to manage the hypertension epidemic.

## Methods

**Fig. 1:**
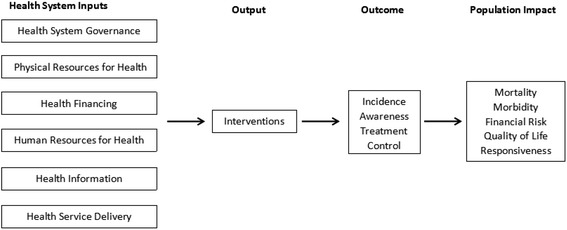
Conceptual framework for health system strengthening and hypertension management

Building on the WHO’s health system monitoring and evaluation framework, and the six building blocks framework [15, 16], we developed a conceptual framework that illustrates how health system factors influence the outcome and impact of hypertension management (Fig. 1). The framework divides the system level inputs into six domains, namely health system governance, physical resources for health, health financing, human resources for health, health information, and health service delivery. We refers health system governance to strategic policy making, system design, effective oversight, coalition-building, regulation, and accountability. We refers physical resources for health to the instruments, equipment and materials to prevent, diagnose, treat, monitor or remit the diseases. For health financing, we use a broad concept incorporating the collection, pooling, and allocation of money to cover the health needs of the people. Thus both the risk pooling and purchasing mechanisms are included. As for human resources for health, we include health workers in different domains of health systems, such as curative, preventive and rehabilitative care services as well as health education, promotion and research. And health information is the information technologies, guidelines which is generated, compiled, analyzed, synthesized and used by health worker for diagnostic or interventional support. Service delivery refers to different strategies and organizational mechanisms to provide quality care. We incorporate all these concepts referring the use of health services in this review: access, availability, utilization, and coverage. We believe that should a country’s health system be strengthened in these six domains, it would turn out to deliver adequate and efficient interventions so as to positively affect the outcomes of hypertension management (i.e. the incidence of hypertensive disorders, and the awareness, treatment and control for hypertension), and lead to the mitigation of the burden of diseases attributed to hypertension (including morbidity, mortality, financial risks, quality of life and financial burden ushered in by hypertension). Such a framework was used in this review in developing searching strategy (Table 1), inclusion and exclusion criteria (Table 2) and building constructs for data extraction.

**Table 1:** Searching strategy

(((((Humans[Mesh]) AND Hypertension[Mesh]) AND China[Tiab]) AND (Case Reports[Publication Type] OR Observational Study[Publication Type] OR Randomized Controlled Trial[Publication Type] OR Comparative Study[Publication Type] OR Evaluation Studies[Publication Type] OR Meta-Analysis[Publication Type] OR Classical Article [Publication Type] OR Corrected and Republished Article[Publication Type] OR Support of Research[Publication Type] OR Health Surveys[Mesh] OR Health Services Research[Mesh])) AND (Delivery of Health Care, Integrated[Mesh] OR Delivery of Health Care[Mesh] OR Health Services Accessibility[Mesh] OR Primary Health Care[Mesh] OR Drug Utilization Review[Mesh] OR Program Evaluation[Mesh] OR Diagnosis[Mesh] OR Self Care[Mesh] OR Health Education[Mesh] OR Equipment and Supplies[Mesh] OR Health Facilities[Mesh] OR Pharmacy[Mesh] OR Pharmaceutical Services[Mesh] OR Health Manpower[Mesh] OR Attitude of Health Personnel[Mesh] OR Health Information Systems[Mesh] OR Health Information Management[Mesh] OR Guideline [Publication Type] OR Healthcare Financing[Mesh] OR Insurance, Health[Mesh] OR Insurance Coverage[Mesh] OR Fees and Charges[Mesh] OR Financing, Government[Mesh] OR Costs and Cost Analysis[Mesh] OR Patient Participation[Mesh] OR Clinical Governance[Mesh] OR Leadership[Mesh] OR Politics[Mesh] OR Health Services Administration[Mesh] OR Health Planning[Mesh] OR Community Health Planning[Mesh] OR Policy[Mesh] OR Health Policy[Mesh] OR Health Priorities[Mesh] OR Health Care Reform[Mesh] OR Health System[All Field] OR National Health Programs[Mesh] OR Social Support[Mesh] OR Social Determinants of Health[Mesh] OR Health Status[Mesh]))

**Table 2:** Inclusion and exclusion criteria

**Inclusion criteria** Studies conducted in Chinese population. Studies that reported the effect of at least one health system factors on the outcome or impact of hypertension management. Outcomes of hypertension management refers to hypertension awareness, treatment and control. Impact of hypertension management refers to mortality, morbidity, financial risks or quality of life that were attributed to hypertension. Studies that reported hypertension prevalence, awareness, treatment or control. Studies that reported any of the following the population impact of hypertension. **Exclusion criteria** Studies not conducted on humankind. Studies conducted outside China. All commentaries, letters and editorials. Studies merely quantify the effects of personal behavioral or modifiable risk factors on hypertension outcomes. Studies that only describe the current knowledge of hypertension, i.e awareness, treatment and control. Studies that merely focus on the population impact of hypertension like mortality, morbidity or financial risk. Studies that only concentrated on community interventions to improve hypertension management without clearly explications of the mechanisms.	

Using the strategy listed in Table 1, we systematically searched the PubMed and China National Knowledge Infrastructure (CNKI). The data were searched from inception to April 13, 2016. Two authors independently screened the search results by title and abstract for eligible studies. And those potentially eligible articles were further screened for inclusion by the full texts. Disagreement were solved by discussions and the inextricable ones were further resolved by the last author. To entrench the data base, references of all the English articles left for full texts analysis were manually screened.

We developed a data extraction form (Additional file 1: Appendix 1), and we built the main constructs for data extraction according to the conceptual framework. Kh Huang, Yt Song and Yh He conducted data extraction under the last author’s supervision. The data were structured by study number, first author, title, published year, study year, study design, setting, sample characteristics, health system factors, the outcomes and the impacts of hypertension management. And quality of the studies were appraised according to their study designs.

Conceptually, studies investigating the effect of health system factors on hypertension management were classified and described according to the framework. We then described the key findings based on the extracted data.

## Results

Five hundred seventy-two publications were identified from PubMed searches and 19 from CNKI, where 93 articles were screened out by title and abstract according to the inclusion and exclusion criteria. Full texts of the 93 articles were obtained and assessed for relevance and ten articles were left according to the inclusion and exclusion criteria for this review. All references of the 10 papers were manually screened and one additional publication were added.

Among the 83 articles excluded, 29 were epidemiological researches purely described the prevalence, awareness, treatment and control of hypertension in China. Twenty-three reported the experiences of community based health promotion programs. These studies were excluded because mechanisms were not disentangled and no efforts were made to link to health system factors. And the other 31 articles were excluded either because the study setting was not in mainland China, or the study was not relative to the outcomes of hypertension management, or the study focused only on mortality, morbidity, quality of life or financial burden attributed to hypertension, or the studies did not provide adequate information.

To describe the current landscape of the outcomes of hypertension management in China, we intentionally extracted information from the 29 epidemiological studies that described the trends in hypertension prevalence, awareness, treatment and control. We combined information from these 29 studies with relevant information from the other 11 papers to describe the current situation for the outcomes of hypertension management in China.

**Fig. 2:**
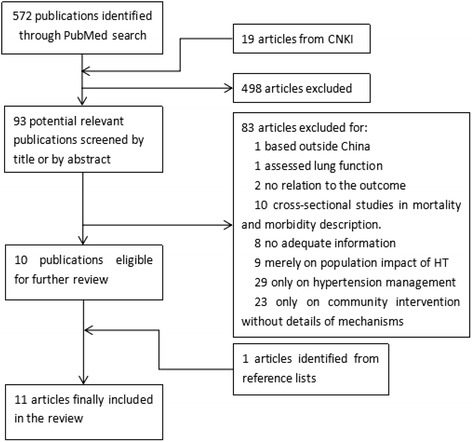
Flowchart demonstrating literature review procedure. *CNKI, China National Knowledge Infrastructure

We finally retained a total of 11 researches conducted in China according to our framework. The study periods ranged from the year 2010 to 2015 (Fig. 2). We appraised quality of the researches according to their study designs. Two of the studies included were randomized control trials (RCTs), three were based on quasi-experimental designs, and six were cross-sectional studies. All these researches were conducted in mainland China, with the study population varies from 213 to 29,411.

### The prevalence, awareness, treatment and control of hypertension

As reported, 27.2 % of the Chinese adults aged 35 to 74 years, representing 129,824,000 persons, had hypertension in 2000 [17]. Whilst 10 years later, based on national data, a prevalence of 40.9 % among people aged 45 years or older was reached [13]. The rapid rising in prevalence seems weird but was corroborated by other sources [13, 17–22]. For example using the WHO’s Study on Global Aging and Adult Health (SAGE) data, Basu et.al. reported a prevalence of 39 % in China during 2007–2010 for people no less than 45 years of age [23]. Whilst with the same data, Lloyd-Sherlock et.al reported a prevalence rate of 59.5 % for Chinese people aged no less than 50 years of age [24]. In summary, hypertension awareness, treatment and control were just 33.7 19.9 and 12.6 % in 1991, but increased to 50.0, 56.1 and 36.4 % in 2012. Although improvement were made in improving its management during the past decade, the rates were still unacceptably low, since the screening and routine management of hypertension has been freely provided in China’s current health reform since 2009 [22, 24–31]. The project was included in national essential public health service to provide the population with health record establishment and blood pressure monitoring. And as for the hypertensives screened out, it required the primary health center to do at least four follow-ups each year [32]. Furthermore, the effective management of hypertension largely becomes a case identification problem since only half of the Chinese people with hypertension are aware of their disorders, whlist majority of the hypertensive individuals who were aware of their hypertension received anti-hypertensive medication and most appeared to be under effective control [13].

### Health system factors associated with good hypertension management

Finally 11 most relevant researches investigating the effects of health system factors on the outcomes or impact of hypertension management were retained. All these identified studies just analyzed the effectiveness of health system factors on hypertension awareness, treatment and control. But none of them intended to link health system factors and incidence of hypertension nor the morbidity, mortality, quality of life or financial burden. One was about health system governance investigating the performance of different organized community health care centers, three were about health financing comparing differences in insurance coverage, three were about health information practicing the hypertension guidelines of China or the WHO, and the rest four were about mechanisms of health service delivery. Unfortunately it was not yet studied for the effects of other two health system domains, namely human resources for health and physical resources for health on the outcomes or impact of hypertension management in China (Table 3).

**Table 3:** Key findings of the eleven most important studies

	Author	Published year	No. and title	Source	Study population	Objective	Key findings
Health System Governance	Wong et al	2012	Performance Comparison among the Major Healthcare Financing Systems in Six Cities of the Pearl River Delta Region, Mainland China.	PLOS ONE	1830 patients of the Pearl River Delta	Compare the effect pf hospital-funded, government-funded and private-funded CHCs	The hypertension treatment rate in hospital-funded community health care centers (CHCs) ranged from 83.1 to 92.1 %, which was lower in government-funded CHCs (70.3 %, adjusted OR 0.46 95 % CI 0.33–0.66) and private-funded CHCs (29.9 %, adjusted OR 0.03 95 % CI 0.02–0.05 ); the control rate in hospital-funded CHCs 22.7 %, ranged from 20.1 to 28.9 %, which was higher in the Government-funded (25.8 %, adjusted OR 1.63, 95 % CI 1.16–2.29), lower in the private-funded CHC (8.9 %, adjusted OR 0.15 95 % CI 0.069–0.31).
Health Financing	Guo et al	2015	The dynamics of hypertension prevalence, awareness, treatment, control and associated factors in Chinese adults.	Journal of Hypertension	75,526 records of 24, 410 adults in 1991–2011 CHNS	Compare effect of insurance with non-insurance	Compared with those without medical insurance, hypertensive patients who had medical insurance were more likely to be aware of their hypertensive condition (aOR 1.5, 95 % CI 1.4–1.7), more likely to be medicated for hypertension (aOR 1.7, 95 % CI 1.5–1.8) and more likely to control their blood pressure within normal range (aOR 1.3, 95 % CI 1.2–1.4).
	Basu et al	2013	Social epidemiology of hypertension in middle-income countries determinants of prevalence, diagnosis, treatment, and control in the WHO SAGE study.	Hypertension	15050 subject in China of SAGA data	Compare effect of voluntary insurance with mandatory insurance	Compared with people covered by mandatory insurance, the risk of being undiagnosed was higher (aOR 4.286, 95 % CI 1.159–15.84, ten times, 95 % CI 2.1–47.4 in Chinese cohort), the risk of being untreated was near four times higher (aOR 4.64, 95 % CI 1.05–20.46), as well as the risk of being uncontrolled (aOR 4.51, 95 % CI 0.98–20.82) .
	Feng et al	2014	Health system strengthening and hypertension awareness, treatment and control:data from the China Health and Retirement Longitudinal Study	Bulletin of the World Health Organization	13,707 individuals of CHARLs	Compare the effect of insurance providing costs of outpatient care with those that contributed nothing to this part	Those with insurance that covered the costs of outpatient care were less likely to be unaware of their hypertension (30.1 % VS 44.4 %, aRR: 0.74; 95 % CI: 0.62–0.88) , to be untreated for it (38.2 % VS 57.8 %, aRR: 0.80; 95 % CI: 0.68–0.93) and to be not controlling it effectively (71.0 % VS 81.0 %, aRR: 0.90; 95 % CI: 0.83–1.00), when compared with those in the insurance contributed nothing to outpatient care.
Health Information	Wang et al	2010	Hypertension Control in Communities (HCC): evaluation result of blood pressure management among hypertensive.	Chinese Journal of Epidemiology	29,411 subjects in six provinces	Evaluate the effect of guideline-based hypertension management	After 1-year intervention, hypertension control rate raised from 21.6 to 74.7 %(*P* < 0.05), with an estimated effect of 53.1 % (95 % CI 52.4–53.8 %).
	Li et al	2015	Effects of guideline-based hypertension management in rural areas of Guangdong Province.	Chinese Medical Journal	3113 patients with essential hypertension in rural Guangdong	Compare effect of traditional therapy and the guideline-based HTN management (the novel therapy)	After 2 years following up, hypertension treatment and control increased in both groups and the control rate increased more significantly in the guideline-based group (from 31.1 to 37.9 % in the traditional group, and from 26.9 to 64.3 % in the guideline-based group, *P* < 0.001).
	Mendis et al	2010	Cardiovascular risk management and its impact on hypertension control in primary care in low-resource settings a cluster-randomized trial	Bulletin of the World Health Organization	2397 patients in China and Nigeria	Evaluate the effect of WHO CVD risk management package	After 12-month following up, the reductions of systolic blood pressure and diastolic blood pressure were significantly greater in the intervention group, with the marginal effects size of 3.86 and 1.53 mmHg in China (−13.28 vs −9.42 mmHg, −6.07 vs −4.54 mmHg, *P* < 0.001).
Health Service delivery	Lu et al	2015	Community-based interventions in hypertensive patients a comparison of three health education strategies	BMC Public Health	360 participants in Guangdong	Compare three health education strategies including self-learning, monthly lecture and interactive education workshop.	Compared with self-learning, after the 2-y intervention, the likelihood of normalized BP was greater in lecture group (from41.2 to 63.2 %, *p* < 0.001, aOR 2.37, 95 % CI 1.26–4.47) and interactive workshop group (from 40.2 to 86.3 %, *p* < 0.001, aOR 14.66, 95 % CI 6.59–32.62 and 2.37).
	Yun et al	2014	Effectiveness of a Community-Based Individualized Lifestyle Intervention Among Older Adults With Diabetes and Hypertension, Tianjin, China, 2008–2009	Prev Chronic Dis	213 participants of five local community health clinics in Tianjin	Evaluate the effect of Zhiji management	Systolic blood pressure and diastolic blood pressure decreased significantly by 10.9 and 4.0 mmHg in the treatment group (*P* < 0.001).
	Niu et al	2014	Differences and determinants in access to essential public health services in China a case study with hypertension people and under-sixes as target population.	Chin Med J (Engl)	1505 hypertensive patients	Evaluate the effect of accessibility	The control rate in those who lived less than 5 min away from the nearest health institution was 39.3 %, slightly higher than the rate for those who lived more than 20 min away, which was 35.8 % (aOR 1.03, 95 % CI 0.60–1.79).
	Gu et al	2015	The Cost-Effectiveness of Low-Cost Essential Antihypertensive Medicines for Hypertension Control in China: A Modelling Study	PLOS Medicine		Evaluate the cost-effectiveness of different treating strategy	Treating all hypertensives for primary and secondary prevention to goal of <140/90 if age 35–64 years, goal of <150/90 if age65 could prevent about 800,000 cardiovascular disease events annually (95 % CI 0.6–1.0 million) and was borderline cost-effective incremental to treating only cardiovascular disease and stage two patients (2015 Int$13,000 per QALY gained [95 % CI, Int$10,000 to Int$18,000])

#### Health governance

Only one cross-sectional study explored the effect of health governance on hypertension management outcomes. Wong et al conducted the study in six cities of southern China in the year 2010 [33]. Using data covering about 1830 hypertensive patients, the study compared performance of hypertension management among three different organized community health care centers (CHCs). The authors found that compared with hospital-funded CHCs, government-funded CHCs had significantly lower prescriptions of anti-hypertensive pharmaceutics with an aOR of 0.46 (95 % CI 0.33–0.66). However hypertension subjects managed by government-funded CHCs were more likely to control their blood pressure adequately (aOR 1.63, 95 % CI 1.16–2.29), while those in privately-funded CHCs were less likely to achieve optimal blood pressure control (aOR 0.15, 95 % CI 0.07–0.31). The authors interpreted that government-funded primary care were less incentivized to prescribe anti-hypertensive agents, and were more willing to implement policies and guidelines. Since the study was institutional based which only recruited hypertensive patients for study, differences in hypertension awareness for population managed by these three types of CHCs were not analyzed.

#### Health financing

All the three papers were based on cross-sectional designs and analyzed the effects of health insurance coverage on the outcomes of hypertension management. The initial attempt was made by Feng et al. [13] in 2014. Using national data from the China Health and Retirement Longitudinal Study (CHARLs), which investigated 17,708 Chinese individuals aged 45 or older, they found that health insurance coverage was the single most important factor associated with good hypertension management in China. As the authors reported, when compared with patients who were members of insurance schemes that cover costs of outpatient care, such as the Urban Employee Basic Medical Insurance Scheme and the Government Insurance Scheme, those who were members of insurance schemes that do not covered costs of outpatient care, like the Urban Resident Basic Medical Insurance Scheme or the New Cooperative Medical Scheme, had nearly 30 % lower awareness (aRR 0.74, 95 % CI 0.62–0.88), 20 % lower treatment (aRR 0.80, 95 % CI 0.68–0.93) and 10 % lower control (aRR 0.90, 95 % CI 0.83–1.00) rates for their hypertensive disorders. Data from other sources strengthened such associations. For example, using data from the China Health and Nutrition Surveys (CHNS), Guo et al. reported in the year 2015 that hypertensive patients who had medical insurance were more likely to be aware of their hypertensive condition (aOR 1.50, 95 % CI 1.40–1.70), more likely to be medicated for hypertension (aOR 1.70, 95 % CI 1.50–1.80) and more likely to control their blood pressure within normal range (aOR 1.30, 95 % CI 1.20–1.40) [22]. And using the SAGE data, Basu et al. found that compared with people covered by mandatory insurance, those covered by voluntary insurance had a ten times greater odds of undiagnosed hypertension (95 % CI 2.10–47.40) in the Chinese sample, and the risk of being untreated was near four times higher (aOR 4.64, 95 % CI 1.05–20.46), as well as the risk of being uncontrolled (aOR 4.51, 95 % CI 0.98–20.82) [23].

#### Health information

The WHO developed a guideline in the World Health Report 2002 using a total risk approach for the prevention of cardiovascular disease [34]. Based on regression models with age, sex, smoking, blood pressure, blood cholesterol, and presence of diabetes as predictors, individuals could be classified into four risk categories of fatal or nonfatal vascular events: very high risk, high risk, moderate risk and low risk. For the individuals of very high or high risk, 3–6 months’ following up for risk monitoring are recommended, 6–12 months’ following up intervals are suggested for those at moderate risk, whilst no follow up were suggested for low risk individuals other than health education on life style modifications. Following up interventions include regular blood pressure monitoring, lifestyle modification consultation like dietary changes, physical activity and weight control, alcohol consumption control, and anti-hypertensive prescription and adjustment. A cardiovascular risk management package was further developed by the WHO later [35]. Main elements includes i) risk-assessment and risk-management algorithms; lifestyle counseling protocols; anti-hypertensive agents’ treatment protocols; referral pathways and follow-up schedules; ii) tools for both providers and patients (training program and manual; self-management tools; personal health records; and tools for improving adherence).

Following the WHO, China already developed its own guidelines called Chinese Guidelines for the Management of Hypertension [36]. The Guideline firstly described the current situation of the increasing prevalence and relatively ineffective management of hypertension in China. And it then recommended a combination of primary, secondary and tertiary prevention, where two strategies were proposed: population strategy and high risk strategy. The population strategy emphasized a healthy lifestyle, health education, community involvement for all citizens; whilst the high risk strategy recommended health examinations and health management targeting high risk population. As for all the hypertensive patients, the Guideline suggested the establishment of personal health record, and community health promotions to improve outpatient attendances and self-management. Since the Chinese guideline was developed based on the WHO’s, they were similar in most parts. However, none risk assessment algorithms were presented in the Chinese guideline.

Three included articles assessed the effects in carrying out such guidelines for hypertension management. A trial by Wang et al. carried out in six provinces in China evaluated the effect of guideline-based blood pressure management [37]. The authors reported that after 1-year intervention, hypertension control rate raised from 21.6 to 74.7 %( *P* < 0.05), with an estimated effect of 53.1 % (95 % CI 52.4–53.8 %). Li et al. did a quasi-experiment in the year 2015 comparing the performance between traditional therapy and the guideline-based hypertension management [38]. The traditional therapy referred to health education on what is hypertension, the potential consequence of hypertension and the risk factors associated with hypertension. After 2 years follow up, the study showed that hypertension treatment and control increased in both groups and the control rate increased more significantly in the guideline-based group (from 31.1 to 37.9 % in the traditional group, and from 26.9 to 64.3 % in the guideline-based group, *P* < 0.001). Mendis et al. conducted a clustered randomized control trial based on WHO cerebrovascular disease risk management package in China and Nigeria in the year 2006 [7]. The study indicated that introduction of the package resulted in a significant increase in the prescribing of anti-hypertensive agents and after 12-month follow up, the reductions of systolic blood pressure and diastolic blood pressure were significantly greater in the intervention group, with the marginal effects size of 3.86and 1.53 mmHg in China (−13.28 vs −9.42 mmHg, −6.07 vs −4.54 mmHg, *P* < 0.001).

#### Health service delivery

Four researches explicated various mechanisms on how to deliver essential health care service to the hypertensive patients properly and efficiently, among which two randomized control trials investigated strategies to improve the efficacy of lifestyle health education practices; and the other two cross-sectional studies, one analyzed the association between access to health care and hypertension management outcomes. And the other one is a model-based study evaluating the gains and cost-effectiveness of implementing total patients management as compare to severe case management.

Lu et al. conducted a community based health education trial in Dongguan City, Guangdong province during 2011–2013 [39]. With a small sample size of 347, the authors compared the performance of three health education strategies in improving the outcomes of hypertension management. It found that regular use of medications to treat hypertension was more frequent when monthly interactive education workshop and monthly didactic lecture were used (92.3 and 79.8 % vs 50.9 %, *P* = 0.001), as compared to the self-learning material distribution strategy. And hypertension control rates were also higher when the two more active health education strategies were used (aOR 14.66, 95 % CI 6.59–32.62 and 2.37, 95 % CI 1.26–4.47 respectively). The other study conducted by Yun et al. in Tianjin in 2014 corroborated the positive effects for such active education strategy [40]. Based on a “Zhiji management”, i.e. health education by friends, it found systolic blood pressure and diastolic blood pressure decreased significantly by 10.9 and 4.0 mmHg in the treatment group (*P* < 0.001).

Niu et al. assessed the association of accessibility of essential public health services and good hypertension management [41]. It was reported that the distance between the nearest primary health care institution and the homes of patients could influence the likelihood to the hypertensives to achieve optimal blood pressure control, where the control rate in those who lived less than 5 min away from the nearest health institution was 39.3 %, slightly higher than the rate for those who lived more than 20 min away, which was 35.8 % (aOR 1.03, 95 % CI 0.60–1.79).

Gu et al. conducted a model-based cost-effectiveness analysis comparing total patients management with severe case management. It was found that treating all hypertensives for primary and secondary prevention to goal of <140/90 if age 35–64 years, goal of <150/90 if age ≥65 could prevent about 800,000 cardiovascular disease events annually (95 % CI 0.6–1.0 million) and was borderline cost-effective incremental to treating only cardiovascular disease and stage two patients (2015 Int$13,000 per QALY gained [95 % CI, Int$10,000 to Int$18,000]) [42]. That means the total patients management strategy seems to be more effective with slightly incremental cost than severe case management.

## Conclusions

The prevalence of hypertension has been rising rapidly in China. Our systematical search found quite a few epidemiological researches on the prevalence, awareness, treatment and control of hypertension in China which had been intentionally described. It was reported that the management of hypertension in China is a detection problem rather than treatment problem. And the efforts of public health community were also abundant, mainly investing in community based health promotion programs. However, less attentions were paid by the Chinese researchers to investigate in the mechanisms that linked various health system factors to the outcome and impact of hypertension management in China, while no evidence was found to clearly link between health system factors and hypertension incidence or its population impact, such as morbidity, mortality, quality of life and financial burden. For system factors, we identified no evidence on the association between human resources for health and physical resources for health and hypertension management. While we only identified paucity of evidence with limited study quality, showing that the improving health system governance, health finance, health information, and health service delivery can make a difference on hypertension management since good governance in community health centers, more generous health insurance covering outpatient care, available guideline directing management and interactive health education strategy as well as total patients management were reported to be associated with good hypertension management. It is therefore strongly called for joint efforts from health system and epidemiological researchers to extend knowledge in this area.

## Additional file

Additional file 1: Appendix 1. (XLSX 408 kb)

## Abbreviations

CHARLs: China Health and Retirement Longitudinal Study
CHCs: Health care centers
CHNS: China Health and Nutrition Surveys
CNKI: China National Knowledge Infrastructure
CVDs: Cardiovascular diseases
LMICs: Low-and middle-income countries
NCDs: Non-communicable diseases
SAGE: Study on Global Aging and Adult Health
WHO: World Health Organization
